# Grant writing and grant peer review as questionable research practices

**DOI:** 10.12688/f1000research.73893.2

**Published:** 2021-12-24

**Authors:** Stijn Conix, Andreas De Block, Krist Vaesen

**Affiliations:** 1 Centre for Logic and Philosophy of Science, KU Leuven, Leuven, Vlaams Brabant, 3000, Belgium; 2 Philosophy & Ethics, Eindhoven University of Technology, Eindhoven, The Netherlands

**Keywords:** peer review, grant review, project funding, research ethics, ethics of funding, science funding

## Abstract

A large part of governmental research funding is currently distributed through the peer review of project proposals. In this paper, we argue that such funding systems incentivize and even force researchers to violate five moral values, each of which is central to commonly used scientific codes of conduct. Our argument complements existing epistemic arguments against peer-review project funding systems and, accordingly, strengthens the mounting calls for reform of these systems.

## 1. Introduction

In industrialized societies, a large fraction of the governmental budgets for research is allocated through competitive peer review of project proposals. This popular mode of funding allocation has been criticized for not delivering the scientific goods it was intended to deliver. Evidence has shown that grant peer review is costly, that the ranking it produces lacks validity, and that it does not promote novel views ( Guthrie, Ghiga, and Wooding 2018; Herbert *et al*. 2013; Link, Swann, and Bozeman 2008 and other references below). But whereas the epistemic defects of peer-review project funding (PRPF) have been extensively studied, its ethical shortcomings have received little scholarly attention. This is surprising, as there has been a strong increase in attention for the prevalence of misconduct and questionable research practices in other parts of science over the past decade ( Xie *et al*. 2021). It is these ethical shortcomings of PRPF that the current paper is concerned with. More specifically, we will argue that PRPF systems prompt behaviour that violates moral values and norms that, according to prominent scientific codes of conduct, scientists, funding agencies and other stakeholders of PRPF are expected to conform to.

As we will see, PRPF systems exert a whole range of different pressures on applicants, reviewers and funders to behave unethically. Importantly, these pressures vary in strength. Sometimes, the pressures are best thought of as (mere) incentives. Those incentives make it more likely that individual researchers will act unethically, primarily because such morally questionable behaviour will increase their chances of success in grant acquisition. Yet, such incentives do not really *require* researchers to engage in unethical practices. On the other hand, there are also what we will call ‘forces’. Because of these forces, researchers who want to apply for research funding or who agree to serve as a grant-decision maker *are required to* behave in a way that most researchers deem ethically questionable.

The conclusion of this paper is that the academic community should reconsider its widespread use of PRPF systems, not just because of these systems’ (alleged) inefficiency and epistemic shortcomings, but also because they almost inevitably promote ethically questionable behaviour.

In the next section, we briefly discuss the background, prominence and epistemic shortcomings of PRPF. The third section analyses prominent scientific codes of conduct (CoC) to identify the ethical values that are supposed to guide the behaviour of researchers. The fourth section then argues that PRPF systems come with a whole range of incentives and forces that prompt violations of these values. Finally, the fifth section discusses what is needed to make the allocation of research funding more ethical.

## 2. PRPF: background, prominence and epistemic shortcomings

Since the 1970s, governments have distributed an increasing fraction of their resources for research on the basis of competition, following a model that the American National Science Foundation (NSF) put in place in the 1950s ( England 1982). These governments urged funding agencies to organize competitions among researchers, such that project proposals are assessed by academic peers, in a way analogous to the peer review of scientific articles.

At present, science funding agencies in industrialized societies allocate much of their funding through PRPF. ^1^ In the US, for example, the National Institutes of Health (NIH) annually invests over $30 billion in basic and applied biomedical research. All of that money is allocated through PRPF. The same holds true for the budget that the NSF spends on basic research (about $8.8 billion per year). ^2^ The European Union, finally, allocated about €60 billion through PRPF between 2014 and 2020 with its Horizon 2020 programme (Schiermeier 2020). Typically, these PRPF funding schemes have very low success rates, making them highly competitive. ^3^

The current widespread reliance on highly competitive PRPF has a series of consequences for researchers. Trivially, peer review of grant proposals influences what kind of research will be done, and who gets to do it. Successful applications also come with prestige, both within and outside the research community ( Coate and Howson 2016). Relatedly, research careers can be made or broken by grant applications. Many research institutions now make tenure, promotions and salary raises dependent on a researcher’s success in prestigious calls ( Dunn, Iglewicz, and Zisook 2020; Joiner and Wormsley 2005) and success in grant funding competitions is one of the most common criteria that institutions use for review, tenure and promotion ( Rice *et al.* 2020). For example, several European universities give a substantial salary supplement to successful ERC-applicants for the duration of their project. In Germany, there is a bonus system for professors that regularly includes targets for grant acquisitions ( Erina 2015).

Given its crucial role in today’s science, one would expect that PRPF reliably and efficiently selects the best scientific projects. Yet, the literature on research funding has raised serious doubts about this. Several studies indicate that peer review is not a very reliable or valid method for evaluating research proposals. Regarding reliability, Kaplan *et al.* (2008), for instance, argue that for the mandated level of precision in reviewers’ scores, the NIH needs as many as 40,000 reviewers per project instead of the 4 reviewers it now aims for. Moreover, biases such as cronyism are pervasive in grant funding ( van den Besselaar 2012; Guthrie, Ghiga, and Wooding 2018).

Since low reliability is typically associated with low validity, it should not come as a surprise that reviewers often fail to predict the scientific success of the proposals they evaluate. For instance, there is no or only a weak correlation between the review score of a project and its eventual bibliometric impact ( Doyle *et al*. 2015; Fang, Bowen, and Casadevall 2016; van den Besselaar and Sandström 2015). Plenty of anecdotal evidence suggests that review panels do a poor job at predicting success: Nobel Prize laureates have repeatedly complained about the difficulties they have experienced in getting their award-winning work past grant review committees ( Bendiscioli 2019; Johnson *et al.* 2010; Kim 2006; Marshall 2005; Taubes 1986).

Other epistemic problems relate to the types of knowledge that are generated by PRPF funded projects. Although many funding agencies emphasize that their aim is to fund innovative research, PRPF systems might instead be conservative, and favour well-established views rather than radically new ideas (see Nicholson and Ioannidis 2012; and reviews by Guthrie, Ghiga, and Wooding 2018; Guthrie *et al.* 2019).

These epistemic problems might be justifiable if the costs and impacts of the system were relatively low. Yet, the costs of PRPF turn out to be very high. Link *et al.* (2008) show that researchers at R1-universities in the United States spend on average more than four hours a week writing project applications. Similar studies were conducted for the National Health and Medical Research Council, a major funding organization for biomedical sciences in Australia. The results of these studies indicate that the time investment in 2009 was as much as 180 years of research time to fund 620 projects, and by 2013, the costs had gone up to more than 500 years of research time – equivalent to € 41 million in salary – to fund approximately 700 projects with a total value of € 226 million ( Herbert *et al.* 2013).

## 3. Ethical values and norms associated with research integrity

Individual ethical behaviour co-varies with situational factors and with personality traits ( Bruton *et al*. 2020). Here, we will be concerned with the role of situational factors, and more specifically with how institutional and structural aspects of research environments prompt unethical behaviour. Our focus on systemic incentives fits today’s scholarship on research integrity. For example, in a recent consensus study report of the National Academies of Sciences-Engineering-Medicine ( NAS 2017, 208), it is noted that “patterns of funding and organization that have emerged over the past few decades in the United States have created environments increasingly characterized by elements [ …] that are associated with cheating, such as very high stakes, a very low expectation of success, and peer cultures that accept corner cutting” ( NAS 2017, 98). We concur, and analyse the characteristics of PRPF that lead to unethical behaviour below. Before doing this, we first identify the core principles of research integrity.

Scientists are bound by a number of science-specific norms and values. These norms and values are frequently made explicit in scientific CoCs. Such CoCs are almost invariably based on, or derived from, the five norms developed by Robert Merton ( Radder 2019). ^4^ CoCs are a good starting point for our analysis because they are the result of extended debates among a wide variety of stakeholders. They function as consensus statements, reflecting an overall agreement among these stakeholders about the norms that should guide research, and present both minimal conditions for ethical research practices and aspirations. In addition, institutions and funding agencies often adopt these CoCs as a framework for their own policies and regulations on research integrity. ^5^ Hence, if we can show that PRPF threatens the core values of the CoCs, we have a strong case for urging these academic institutions and funders to revise their policies on PRPF.

We analyzed the following five CoCs: the *Singapore Statement on Research Integrity* ( Resnik and Shamoo 2011) that was written at the second World Conference on Research Integrity, the *European Code of Conduct for Research Integrity* (2017), developed by the European Science Foundation and All European Academies, *Doing Global Science: a guide to responsible conduct in the global research enterprise* ( IAP 2016) written by a committee of leading scholars on research ethics, *Fostering Integrity in Research* ( NAS 2017), a consensus document published by the National Academy of Sciences, and *Ethical Guidelines for Peer Reviewers* ( COPE 2013), drafted by the Committee on Publication Ethics. These CoCs were selected because of (1) their geographical focus (EU, USA, world), (2) their generality (not about one discipline or aspect, but about science in general), and (3) their authoritative status within the scientific community. We also analyzed *Ethical Guidelines for Peer Reviewers* ( COPE 2013), because peer review is central to PRPF.

The selected CoCs differ somewhat in their definitions of integrity and misconduct. They also vary in their approach. Some CoCs are more value-based, and state which values (e.g. honesty) should guide research. Other CoCs are more norm-based, and primarily indicate which behaviours need to be sanctioned or stimulated ( Godecharle, Nemery, and Dierickx 2014). The CoCs also vary with regard to the values and norms they include. This variation is largely due to the inevitable vagueness associated with the formulation of values and norms, and due to the different objectives of the CoCs. Still, the differences do not reflect deep disagreements on what constitutes misconduct or about the core values.

In order to facilitate our assessment, we have extracted from the CoCs one list of values and norms. This list ignores the subtle differences just mentioned, and captures what the CoCs have in common. Table 1 summarizes how our list maps onto the values prioritized by the various CoCs.

**Table 1.** Mapping of the new list of values (shaded grey) onto Codes of Conduct (CoCs, shaded black). The CoCs are, in order, the European Code of Conduct for Research Integrity (ECCRI), Doing Global Science (DGS), Fostering Integrity in Research (FIR), the Singapore Statement on Research Integrity (SSRI) and Ethical Guidelines for Peer Reviewers (EGPR).

	ECCRI	DGS	FIR	SSRI	EGPR
Accountability	Accountability	Reliability, Accountability	Openness, Accountability	Accountability	Accountability, Professional responsibility
Honesty	Reliability, Honesty	Honesty, Objectivity, Reliability, Skepticism, Openness	Honesty, Openness	Honesty	Honesty, Professional responsibility
Impartiality	Reliability, Honesty	Fairness, Objectivity, Scepticism	Objectivity, Fairness	Professionalism	Honesty, Accountability, Being unbiased
Responsibility	Respect		Stewardship	Stewardship	
Fairness	Honesty, Respect	Fairness, Openness	Fairness, Stewardship	Professionalism, Stewardship	Accountability, Confidentiality

Our list comprises the following values and norms:

• *Accountability* entails that scientists should be able to explain and justify their claims and actions.
• *Honesty* obliges scientists to be accurate, transparent and clear in all their communication. Researchers violate this value when they fabricate or falsify data, when they present findings in a misleading way, and when they are insufficiently open about the uncertainty of their claims.
• *Impartiality* means that researchers do not let their personal opinions, interests, preferences prejudices or the interests of the bodies commissioning their work influence their decisions and judgements. Rather, researchers’ decisions and judgements should serve the aims of science (e.g., truth, instrumental value).
• *Responsibility* requires researchers to take into consideration the broad interests of society. Researchers should spend their resources on research that benefits society, that does not violate the ethical guidelines for activities involving human subjects and animals, and that properly mitigates possible harms and risks.
• *Fairness* implies that scientists should show due respect to everybody they interact with in a scientific context, and sufficiently acknowledge the work of others. This applies to interactions with fellow-scientists, but also to interactions with participants in experiments, the readers of scientific publications, administrative staff, students and funders.

We acknowledge that there is some overlap between some of these values (as there is in the CoCs) and that alternative categorizations are possible. For example, the problem of double dipping (i.e. researchers using the same project in different funding schemes) is as much a violation of norms like solidarity or generosity as it is a violation of honesty. Thus, an alternative classification that includes solidarity and generosity would be viable as well. Still, our categorization fits our purposes, as it captures the main ways in which PRPF invites ethically questionable practices, and helps us (in Section 4) in structuring them.

Importantly, on a final note, in the remainder we will construe the five values of research integrity primarily as *ethical* values. We acknowledge, though, that each of them is directly or indirectly related to epistemic values. Consequently, many of the problems we discuss are epistemic as well as moral problems. It is no coincidence that this is so: research integrity is concerned with scientific research, and society values such research primarily because of the epistemic goods it delivers. Given this entanglement of ethical and epistemic considerations, our focus on the ethical aspects of PRPFs is inevitably somewhat artificial. However, such a focus is useful here, as it more clearly brings out an important point that has not received due attention: the ethical dimension of the problems that PRPF systems give rise to. In any case, to the extent one regards these values as merely epistemic, our paper complements the epistemic worries about PRPF raised and reviewed in Section 2.

## 4. PRPF prompts violations of research integrity

This section argues that PRPF forces or incentivizes researchers to violate each of the five aforementioned values. Many ethically questionable practices can be categorized as a violation of more than one value. When that is the case, we just place and discuss it under one value. Our focus will be on ethical problems that arise for *individual researchers* who apply for, evaluate and receive research grants. System-level moral issues, such as the inefficiency of PRPF systems, which are primarily associated with policy-makers and funding organizations, fall outside our scope.

Before we turn to these practices, it is good to note that most of them have not, or only rarely, been studied. We refer to empirical work whenever it exists, but this is unfortunately not the case for all practices. Even though this means that there is not always published evidence that backs up our claims that these practices are prevalent, we believe that most people familiar with academia and its funding processes will recognise the practices we discuss and know how common they are. We too have at some point engaged in some of these practices, and we expect that most readers of this paper also either engage in them or know of colleagues who do.

### 4.1 Accountability

Scientists are bound by the norm of accountability: they should only make claims that are justified to the degree that is appropriate for the context in which they make these claims. As both funding applications and review reports consist in claims about future research, this norm is directly relevant to the way PRPF distributes research funding. We argue that PRPF commonly forces both applicants and reviewers to make claims they cannot sufficiently justify.

First, consider applicants. Most grant applications require applicants to develop detailed timelines, and to describe expected milestones, results and applications. However, the outcomes and course of scientific research are notoriously difficult to predict ( Carrier 2008; Mallapaty 2018; Sinatra *et al*. 2016). Indeed, scientists have been quite wrong about the future impact of, among others, Mendelian genetics, Pasteur’s fermentation theory, continental drift, the idea of Australopithecus being ancestral to Homo, the prion theory (concerning the causes of BSE or “mad cow disease”), and bacterial infection as the cause of stomach ulcers ( Benda and Engels 2011; Gordon and Poulin 2009). Because making predictions of future success or listing project deliverables is a mandatory part of project applications, researchers are thus *forced* to make claims that they cannot sufficiently justify. Note that some projects (viz., risky ones) might be subject to this worry more than others. But even the success of allegedly fail-safe projects depends on various factors that are not under the control of the researchers who write the grant applications, including, among others, fluctuations in the supply of qualified labour, political and economic developments, changes in institutional policies, contingencies in the poorly understood process from invention to innovation, and personal and inter-personal issues arising within the project team.

Grant-decision makers (grant committee members and peer reviewers), too, are forced by PRPF to make claims they cannot justify. Note that their decisions require a high degree of justification, as they decide over large amounts of money and their decisions have a great impact on the careers of researchers, the course of science, and the people potentially affected by the outcomes of the proposed research. One reason why it may often be impossible for grant-decision makers to meet the required high degree of justification is that in many funding competitions there are far more high- quality applications than there is money to distribute. Because scientific success is difficult to predict (see above), grant-decision makers lack grounds for choosing between these high-quality applications ( Kaplan *et al.* 2008). Because of this, there is a push to generate unjustified reasons and to overemphasize tiny or even insignificant differences between granted and rejected proposals. Another reason why the required degree of justification is rarely met is that grant-decision makers typically do not get all the relevant information that is needed to make a proper judgment. For example, grant-decision makers are often asked to give scores to applicants but due to differences in experience and context are likely to work with a different reference class (e.g., an applicant might be judged top-5% by one reviewer, but top-20% by another because the reviewers come from different fields). In addition, grant-decision makers often have to evaluate projects that fall outside their direct area of expertise. This is the case, for instance, when they serve in interdisciplinary grant committee panels ( Bromham *et al.* 2016).

In light of the above, grant-decision makers are *forced* to make unjustified evaluations. In addition to these forces, there are also various incentives that give rise to violations of accountability. For instance, the large review burden and time-pressure of PRPF may incentivize some reviewers to deliver low-quality reports, and, hence, to make judgments that are insufficiently justified ( Publons 2019). Reviewers in grant panels typically have to read thousands of pages of applications, review reports and researcher profiles. Even the most diligent among them are unlikely to have the time to thoroughly read all these materials. This means they either have to skim through projects, or select a few that are closest to their expertise. In this light, it is also no surprise that reviewers admit that irrelevant factors, such as spelling errors, play a role in their grant decisions ( Inouye and Fiellin 2005; Porter 2005).

### 4.2 Honesty

Norms of honesty demand researchers to not intentionally make false claims. Some indirect implications of these norms are that researchers should not withhold crucial information, include irrelevant information, or use other methods of deception. In the context of research funding, this norm is primarily relevant for project proposals and evaluation reports.

First, PRPF systems strongly incentivize researchers to violate authorship norms. Because of low success rates, the increasing dependence of academic institutions on external grant acquisition, and the prestige derived from successful applications, scientists are strongly encouraged (or obliged by their institutions) to take part in as many funding competitions as possible ( Fang and Casadevall 2016). Because the applicant’s profile plays an important role in the evaluation of grant proposals, senior scientists are most likely to be successful. However, senior scientists rarely have the time to write (many) grant applications. Accordingly, they may be tempted to delegate some or even most of the work of grant writing to their junior staff, and submit the application under their own name. At the same time, there are plenty of funding schemes for which junior researchers (e.g., PhD students, postdocs) are not eligible, even if these schemes are primarily used for funding work carried out by such junior staff (e.g. postdocs and PhDs hired on a project). To the extent that junior researchers contribute to writing such grants, the eligibility criteria of PRPF systems induce them to write applications under a different name. Relatedly, it is a public secret (although how pervasive it is has not been investigated yet) that junior researchers sometimes submit proposals under their own name that they have not written themselves. Grants for junior researchers are then used to pursue the research goals of others (senior researchers, labs). Such practices violate the norm of honesty in that the work of the actual author(s) is not acknowledged, and this for the purpose of deceiving grant-decision makers.

An *incentive* to withhold crucial information is the risk that reviewers will steal the ideas of the applicants they are assessing—and there is usually plenty of time for this to happen, given the typically substantive time delay between application and funding decision. Accordingly, it is no surprise that applicants have characterized their own application strategy as follows: “you only show them [reviewers] enough to get it [your project] funded”, otherwise they will “kill your grant, and then take and do it” (interviewee in Anderson *et al*. 2007a, 425).

Another salient incentive for PRPF systems to be dishonest relates to so-called ‘grantsmanship’. This term generally refers to the art of writing successful funding applications, but is typically used to single out those aspects of the application that are not scientific but rather formal, stylistic and rhetorical. Indeed, many guides of grantsmanship emphasize that grants are in the first place pieces of advertising (e.g., Koppelman and Holloway 2012; Rasey 1999). Because the review process should primarily evaluate scientific merit (rather than formal or stylistic qualities), grantsmanship adds noise to the evaluation system. Such noise is particularly harmful because funding competitions are a zero-sum game: successful applicants win *at the expense of* other applicants. Superior grantsmanship may thus push equally good or better applicants below the funding threshold. Because it is unlikely that reviewers are fully insensitive to factors that are unrelated to scientific merit ( Inouye and Fiellin 2005; Porter 2005), PRPF systems plausibly *reward* grantsmanship. This is illustrated by the staggeringly high success rates of some grant writing consultants: ^6^ being supported by people with no background in the proposed research dramatically increases the chances of getting money for that research.

Another practice that PRPF incentivizes concerns ‘double-dipping’, the practice of submitting the same research project in multiple funding calls without proper acknowledgement. The reasons researchers are incentivized to engage in double-dipping have been mentioned above: low success rates, academic institutions’ increasing dependence on external funding, prestige and so forth. That double-dipping is common is suggested by Garner *et al.* (2013). In their study of U.S. funding in the biomedical sciences, these authors found that, between 2007 and 2011, over $20 million was allocated to projects that had already attracted funding before. Although this amounted to only a small percentage of the total budget that was distributed, Garner *et al.* (2013) suggest it probably is an under-estimation, given the difficulties in finding duplicates. In any case, it *is* research money that cannot be spent on other research projects. Double-dipping includes several dishonest practices, such as, withholding relevant information, self-plagiarism and, plausibly, the use of grant money for purposes other than those for which it was intended.

Finally, PRPF also incentivizes the dishonest practice of applying with research that has already partially been done ( Anderson *et al*. 2007a, 448). In a longitudinal study of grant applications from the *Deutsche Forschungsgemeinschaft*, Serrano Velarde (2018) observes that decreasing success rates have made applicants increasingly concerned with portraying their research projects as certain to be successful. Arguably, they share this concern with funding agencies, that, in light of the demand for greater public accountability, typically ask applicants to specify clear, demonstrably feasible and measurable targets (deliverables, outputs, milestones) ( Frodeman and Briggle 2012). Reviewers, too, seem biased towards success, for they appear to reward projects that are highly likely to achieve what they promise ( Inouye and Fiellin 2005). Because of this, portraying an ongoing or finished research project as if it is merely a research plan, is an effective and – according to interviewees in Anderson *et al.* (2007a) – popular strategy. In that light, it is not surprising that 27% of early-career scientists and 72% of all midcareer scientists in a survey admitted to improper use of funds such as using money from one project in another ( Anderson *et al*. 2007b).

### 4.3 Impartiality

Impartiality means that researchers should aim their decisions and judgements to primarily serve the interests of science. Accordingly, their decisions and judgements should not be led by prejudices, the interests of their sponsors, or any other bias.

There are at least two senses in which PRPF schemes *force* researchers to violate norms of impartiality. First, there is solid evidence that the judgements of grant-decision makers are subject to various biases ( Boudreau *et al*. 2016; Guthrie *et al*. 2019; Nicholson and Ioannidis 2012; van den Besselaar 2012). Thus, at least given the way that PRPF schemes are currently set up, serving as a reviewer presently means engaging in a practice that is known to violate norms of impartiality. Surely, full impartiality is too stringent a demand for many scientific activities—for instance, such a demand would make carrying out research virtually impossible. But in the case of distributing research money, there *do* exist alternatives that fare much better than PRPF when it comes to impartiality (e.g., lotteries, egalitarian sharing, see Section 5).

A second sense in which PRPF schemes force researchers to transgress norms of impartiality relates to the political context that the schemes operate in. In some cases, grant decision-makers might be requested to take into account such things as the geographical and institutional distribution of the grants they award and the political sensitivities of the governments they work for. Hegde (2009) and Batinti (2016), for instance, found that working in a U.S. presidential swing-voter state or in a state of certain congressional appropriators increases applicants’ likelihood of success up to 10.3%. Grant-decision makers thus seem to be compelled to let the interests of the bodies commissioning their grant reviewing work interfere with their judgment. As a result, projects might get funded that are optimal in political terms, but sub-optimal in scientific terms.

Turning to the *incentives* to act against the norms of impartiality, the track record of applicants is an important consideration in grant-decision making. ^7^ Together with the pressure to be successful in grant applications, PRPF might thus indirectly invite applicants to engage in practices that boost their publication record (in terms of number of publications, citation counts, or journal impact factors) but fail to serve the interests of science ( Bouter 2015; Tijdink, Verbeke, and Smulders 2014). Furthermore, to reduce the workload of grant committee members, many PRPF schemes allow applicants to indicate potential reviewers of their proposal; applicants thus get the opportunity to increase the likelihood of receiving a favourable review ( Severin *et al.* 2019). They can further increase this likelihood by selective citing (e.g., citations of possible reviewers, no citations to hostile reviewers), which, according to a survey with experts on research integrity, occurs relatively frequently ( Bouter *et al.* 2016). Similar incentives to violate the norm of impartiality also arise because, unlike reviewers, grant-decision makers will often come from the same country as applicants. For example, up to two thirds of all panel members for the Flemish Research Council (FWO ^8^; Belgium) can have an appointment at a Belgian University. ^9^ Especially in smaller countries like Belgium, these decision makers often participate in applications of their friends (or enemies) and favourite (or disliked) colleagues. Under these circumstances, cronyism is to be expected ( van den Besselaar 2012).

### 4.4 Responsibility

Norms of responsibility require researchers, in their work, to take into account the broad interests of society. This norm is particularly salient in the case of publicly funded research.

Being responsible to taxpayers implies that the returns of public funding bodies’ investments should be public. However, the 1980 Bayh-Dole Act established a legal framework in the U.S. that encourages recipients of public research money to derive patents from their publicly funded research results ( Rai and Sampat 2012). The increasing emphasis that public funding agencies’ place on valorization drives scientists towards research that directly creates economic returns ( De Jonge and Louwaars 2009), and to exploit the commercial opportunities created by the Bayh-Dole Act. Proponents of the act (and of academic patenting more generally) point out that academic patenting benefits society because it promotes commercial development of otherwise purely academic knowledge. Their arguments have been repeatedly criticized on empirical and epistemic grounds ( Mirowski 2011; Radder 2019; Sterckx 2010). But on whatever side of the debate one stands, academic patenting does push publicly acquired knowledge out of the public domain. So at least in this sense, U.S. funding agencies incentivize practices that go against public interests. The same holds true for funding agencies that work for governments that have adopted (viz., Japan) or are in the process of adopting (viz., the E.U.) Bayh-Dole-type legislation ( Lynskey 2006; Mirowski 2011).

Another worrisome practice that researchers participating in PRPF schemes are strongly encouraged to engage in pertains to hiring. PRPF schemes are by definition project-based, and thus only provide funding for the duration of the project. Accordingly, grantees are pressurized to hire, as project collaborators, cheap *temporary* staff (PhD students, Postdocs), even if that staff carries out work that, in the long run, would in a more cost-effective way be carried out by specialized, permanent staff. The considerable costs for society do not stop there: the reliance of grantees on temporary labour presumably also contributes to the mismatch between the production of PhDs and the availability of jobs in the academic sector that industrialized societies are currently facing ( Gould 2015). Although part of the costs of this mismatch can be compensated for by the training that temporary grants may provide for jobs outside academia, the mismatch probably also exacerbates many questionable research practices ( Smaldino and McElreath 2016).

Weaker, but still significant incentives relate to project budgets. For one, various *incentives* invite applicants to apply for more research money than needed, including minimum and maximum budget clauses in grant applications, a lack of institutionalized differentiation with respect to grant size between resource-intensive and less resource-intensive disciplines (for all of these see e.g. the FWO), and pressure from researchers’ home institutes. Further, funds are typically to be spent within the intended timeframe of the project in question; it is an open secret that many researchers are tempted to use, before the actual end of their project, estimated surpluses for purposes unrelated to the proposed research. As Brennan and Magness (2019) put it in their *Cracks in the Ivory Tower*: “If we are not rewarded for being frugal, we might as well [...] buy the nicest computers and hotel rooms our budget permits”.

### 4.5 Fairness

The value of fairness requires that scientists treat everybody they interact with or affect in their work with due respect. Fairness concerns all interpersonal relationships in science, and in that sense overlaps with the other values on our list. Many of the practices we have already described under the headings of honesty, responsibility and objectivity also constitute a lack of respect for other scientists.

In addition to these, at least one other violation of fairness deserves mentioning. Most of the incentivized practices we have discussed here are commonly accepted in academia. For example, researchers write guides about grantsmanship ( Koppelman and Holloway 2012; Rasey 1999), researchers are often expected to apply for more funding than they can effectively use, and at least at our home institutions the use of grant writing consultants is explicitly encouraged. We know of several postdoctoral researchers who are funded by one grant to allocate more than half of their research time to applying for other lab-level research grants. Such violations of norms of research integrity appear to be tolerated by the scientific community. Indeed, it is unlikely that commissions for research integrity would seriously investigate allegations of misconduct if the misconduct consists merely in grantsmanship or ill-justified timelines in applications.

The fact that such ethically questionable practices are widely tolerated is problematic in several ways. First, it is unfair towards those researchers that do not give in to the pushes of PRPF systems to engage in such practices. Second, it is a direct violation of the CoCs, which explicitly warn against tolerating unethical behaviour. Finally, it establishes an unhealthy research culture that makes it difficult for researchers that are not well-established, like junior scholars, to adhere to the CoCs ( Roumbanis 2019).

## 5. Discussion and conclusions

The violations we have listed are not meant to be exhaustive, and they are unlikely to capture all the senses in which PRPF systems prompt ethically questionable research practices. In addition, some may find that most of the questionable research practices we discussed are minor issues, and it is true that none of the problems presents a clear knock-down argument against PRPF. Still, we have seen that PRPF systems force or incentivize researchers to violate, in one way or the other, each of the five norms and values commonly associated with research integrity and included by all major CoCs. And while many of these may seem like only minor violations of the COCs, some, such as double-dipping, self-plagiarism and ghost authorship, are unquestionably problematic and serious. Listing these minor and major violations side by side shows that ignoring these problems comes at a substantial ethical cost, as the issues are numerous, pervasive and unlikely to disappear on their own. In this concluding section, we briefly consider three options for reform.

The first option is to mitigate the perverse incentives associated with PRPF by eliminating or modifying those features of PRPF that prompt questionable behaviour. For instance, funding agencies could remove the demand to formulate strict timelines that indicate expected successes and measurable targets (e.g., number of papers, targeted journals, milestones). Alternatively, they could formally ask reviewers to estimate their confidence in their own reviews, and applicants to disclose any other funding schemes they have applied to with the same project. While making such changes would undoubtedly solve some of the issues we have discussed, this option will presumably remain sub-optimal. To start, many of the features of PRPF were introduced for good reasons. For example, measurable targets make it easier for reviewers to evaluate the output of the project. Second, the likely impact of some of the changes would be limited. If a funding body no longer required applicants to formulate milestones, applicants would arguably continue mentioning them, because they intuit that milestones strengthen their application. Similarly, reviewers’ estimates of uncertainty would also be subject to uncertainty, thus propagating the same problem on a different level. Third, some of the incentives we have discussed seem to be intrinsic to PRPF systems and cannot be substantively modified without abandoning PRPF altogether. For instance, as long as grant decision-makers are human, the norm of impartiality will be hard to conform to.

A second option is to draft regulations for the specific context of PRPF— a ‘CoC for grant writing and reviewing’—and to implement mechanisms for the enforcement of those regulations. Some funding agencies have already set steps in this direction. The Flemish and Dutch research councils, for example, require applicants in many of its funding schemes to indicate whether they have submitted or plan to submit their proposal with other funding agencies. It remains to be seen whether such measures will effectively reduce the prevalence of the practices that they target, such as double dipping. But, in any case, it is doubtful that regulatory work will be enough to address all the worries that we have raised. Indeed, as we have seen, PRPF systems are still subject to cronyism, in spite of codes of conduct that explicitly disapprove of cronyism ( van den Besselaar 2012).

A final, more radical option is to put into effect alternative allocation systems. Various such systems have been proposed, primarily with the aim of addressing the epistemic shortcomings of PRPF ( Guthrie 2019). These alternatives include peer-to-peer distribution ( Bollen *et al.* 2017), allocation on the basis of past performance ( Bolli 2014; Roy 1985), a (modified) lottery among short project proposals ( Fang and Casadevall 2016), bicameral grant review ( Forsdyke 1991), using AI for peer review ( Checco *et al.* 2021), and baseline funding ( Vaesen and Katzav 2017). Of these alternatives, bicameral grant review, allocation on the basis of past performance and peer-to-peer distribution are most similar with PRPF and, in that they still involve (serious) competition among applicants; accordingly, they most likely share PRPF’s shortcomings. Relying on AI for peer review to increase fairness and decrease costs is a promising alternative, but does not seem possible yet. More research on this topic is needed, in particular to ensure that avoiding human biases does not come at the cost of introducing algorithmic biases ( Checco *et al.* 2021). The two other alternatives, i.e. lottery-based systems and baseline funding, seem more promising with respect to research integrity. While they might suffer from different (unforeseen) moral problems, they seem less sensitive to many of the issues discussed in this paper. This is because they differ from PRPF in three crucial respects.

First, baseline funding and lottery-based systems substantively minimize reliance on judgements that we have seen to be problematic. These judgements include unjustifiable predictions in applications and review reports (violation of accountability), omitting crucial methodological details in project proposals (violation of honesty), and biased grant evaluations (violation of impartiality).

Second, baseline and lottery-based systems are relatively difficult to game. This is because they disregard many of the allegedly salient, but easily manipulated, differences among applicants that PRPF grant decisions are informed by. For instance, baseline and lottery-based systems are largely immune to grantsmanship and do not reward the many questionable practices that applicants might use to pimp their publication track record (e.g., salami-slicing, cutting corners, plagiarism, not publishing negative results).

Finally, the credit and prestige that applicants derive from a baseline or lottery-based grant would, relative to the credit and prestige derived from a PRPF grant, be minor or even nil. Indeed, there is little merit in acquiring a grant based on chance (lottery-based) or when every researcher gets one (baseline funding). An additional benefit of decoupling (alleged) merit and funding is that it would temper the Matthew effect and the overconfidence associated with repeated success in PRPF competitions. A non-merit based system would promote intellectual humility, a value that is both epistemically and ethically desirable ( Alfano, Tanesini, and Lynch 2020).

A way to summarize these three differences between PRPF and the two alternatives is that only the former distributes funding on the basis of *competition* between researchers. In various ways, the competition for funding incentivizes researchers to cut corners and violate generally accepted norms of research integrity. This is interesting, as competition also seems to incentivize researchers to violate CoCs in other parts of science such as the research process and journal publications ( Anderson *et al.* 2007a; Fang and Casadevall 2015; Fanelli 2010; Tijdink, Verbeke, and Smulders 2014). Our paper thus adds to these existing arguments for making science and its funding less competitive. Moreover, given PRPF’s epistemic shortcomings, and the likely epistemic advantages of baseline and lottery-based funding, there are also non-ethical reasons to take these alternative systems seriously.

## Data availability

No data are associated with this article.

## Footnotes

^1^
Throughout this paper, we use the term ‘science’ to refer to all academic disciplines.

^2^
These numbers are for 2020, see https://www.nih.gov/grants-funding and https://www.nsf.gov/about/glance.jsp

^3^
This high degree of competition is not inherent to PRPF, and it is perfectly possible to use PRPF with moderate or low levels of competition. As it is our aim to evaluate PRPF as it currently functions, we evaluate the system in its current highly competitive form.

^4^
This is not to say that the Mertonian norms haven’t been criticized (See Knuuttila 2012 for an overview). For one, there seems to be a disconnect between the norms and actual scientific practice, especially in the context of commodified science. In this sense, the ethos of science conflicts with the organization of science. In fact, our arguments about PRPF illustrate that the organization of science indeed is difficult to bring into accord with the regulative ideals formulated by Merton.

^5^
See e.g. the list of stakeholders in the European Code of Conduct for Research Integrity (2017).

^6^
These success rates are advertised on the websites of grant writing consultants. We do not refer to specific websites here, because we think some of their practices are unethical.

^7^

See e.g. ERC ( https://ec.europa.eu/research/participants/data/ref/h2020/wp/2018-2020/erc/h2020-wp18-erc_en.pdf#page=34) and NSF ( https://www.nsf.gov/pubs/policydocs/pappg18_1/pappg_3.jsp#IIIA2a).

^8^

https://www.fwo.be/en/fellowships-funding/research-projects/junior-and-senior-research-projects/

^9^

https://www.fwo.be/nl/het-fwo/organisatie/fwo-expertpanels/reglement-fwo-interne-en-externe-peer-review/
