# Supplementary material for: Grant writing and grant peer review as questionable research practices
Source: F1000Res. 2021 Dec 24;10:1126. Originally published 2021 Nov 8. [Version 2] doi: 10.12688/f1000research.73893.2 (PMC8825646; doi:10.12688/f1000research.73893.2)
Supplement: Peer review history

# Peer review: Reviewer response for version 2

**DOI:** 10.5256/f1000research.79975.r118342

**Published:** 2022-01-04

**Authors:** Lambros Roumbanis

**Affiliations:** 1 Stockholm Centre for Organizational Research (SCORE), Stockholm University, Stockholm, Sweden

Thank you for the responses on my comments. I think you managed to revise your already very interesting and well-written article into a really fine contribution to the literature on peer review and research evaluation.

Is the topic of the opinion article discussed accurately in the context of the current literature?

Yes

Are arguments sufficiently supported by evidence from the published literature?

Yes

Are all factual statements correct and adequately supported by citations?

Yes

Are the conclusions drawn balanced and justified on the basis of the presented arguments?

Yes

Reviewer Expertise:

Sociology of Science; Science and Technology studies (STS); Evaluation studies; Theory of Science; Sociology of Judgment and Decision-making.

I confirm that I have read this submission and believe that I have an appropriate level of expertise to confirm that it is of an acceptable scientific standard.

# Peer review: Reviewer response for version 2

**DOI:** 10.5256/f1000research.79975.r118341

**Published:** 2022-01-04

**Authors:** Tamarinde Haven

**Affiliations:** 1 Charité, Berlin, Germany

The authors have sufficiently addressed my comments, the paper now reads (even more) nuanced and presents various new topics of interest for the scientific community at large.

Is the topic of the opinion article discussed accurately in the context of the current literature?

Partly

Are arguments sufficiently supported by evidence from the published literature?

Partly

Are all factual statements correct and adequately supported by citations?

Yes

Are the conclusions drawn balanced and justified on the basis of the presented arguments?

Partly

Reviewer Expertise:

My area of interest is research integrity, in addition, I have a background in philosophy, psychology and epidemiology.

I confirm that I have read this submission and believe that I have an appropriate level of expertise to confirm that it is of an acceptable scientific standard.

# Peer review: Reviewer response for version 1

**DOI:** 10.5256/f1000research.77582.r99420

**Published:** 2021-11-16

**Authors:** Tamarinde Haven

**Affiliations:** 1 Charité, Berlin, Germany

**Summary:**

 The authors argue that rather typical behaviours from applicants as well as reviewers in peer-review project funding (PRPF) clash with key values from research integrity codes of conduct (CoCs). After teasing out 5 key values that are shared by a varying set of CoCs, they show for that each value is harmed in the current PRPF system. I find it interesting that the authors focus on questionable research practices by the applicants as well as the reviewers, they present some behaviours that might be familiar to readers, but their interpretation of these behaviours as questionable because they harm the values in CoCs is thought-provoking.

**To the questions below:**

- Is the topic of the opinion article discussed accurately in the context of the current literature?

There is an increasing amount of research that investigates questionable research practices, a recent systematic review might provide some points the authors can connect to: Xie, Y., Wang, K., & Kong, Y. (2021). Prevalence of Research Misconduct and Questionable Research Practices: A Systematic Review and Meta-Analysis. Sci Eng Ethics, 27(4), 41.

- Are all factual statements correct and adequately supported by citations?

When the statements are not supported by citations, e.g., p. 5, paragraph 3, the authors acknowledge this, which is normal practice for a philosophical paper.

- Are arguments sufficiently supported by evidence from the published literature?

Not all, but the authors explicitly acknowledge this. That said, their argumentation for this can be substantiated at places, which I point to below.

- Are the conclusions drawn balanced and justified on the basis of the presented arguments?

Most of the authors’ main conclusions are balanced and justified, but I find part of their concluding statements unjustified. In particular, “In fact, our assessment includes many of the ‘cardinal sins’ against research integrity: self-plagiarism (in the form of double-dipping), taking credit for someone else’s work (in cases where junior researchers write applications for their senior colleagues) and, potentially, falsification and fabrication (in cases where scientific results are adjusted to conform to promises made in the grant application).” p. 8 seems unjustified. The ‘cardinal sins’ indeed include plagiarism, but not *self*-plagiarism, which in various CoC is explicitly separated from plagiarising others, see e.g., Dutch CoC. Taking credit for someone else’s work is guest authorship, which is related to plagiarism but often not considered a cardinal sin. In the article, the authors talk about making claims in grant applications that the grant requesters may not be able to justify, but they don’t go in-depth into the pressure put on researchers *after* having received a grant, where these cases of potential falsification and fabrication should then supposedly happen. Whether the successful applicants do this seems an empirical claim to me, and one that seems rather unlikely.

**Suggestions:**

 I have three suggestions. First, a substantial portion of the critique seems focused on the competition that is inherent in peer-review project funding, not so much the peer-review project funding system per se. The competition seems to do the heavy lifting in increasing the likelihood of researchers or reviewers engaging in questionable behaviour (at least for parts of Accountability, Honesty and Impartiality). I am aware that the authors acknowledge how PRPF is a case of organised competition, but it seems to me that PRPF in a situation of medium competition (instead of contemporary excessive competition) would be associated with less questionable behaviour or would at least be a much weaker incentive. It would be helpful to read how the authors see this, because the topic of competition has been discussed by various research integrity scholars (a great example is the paper by Anderson and colleagues from 2007).

 My second suggestion is that although the authors acknowledge the inevitable overlap of epistemic and moral norms, the harm resulting from the questionable behaviours (QRPs) described seems primarily epistemic. I wonder why the authors hold on to framing their arguments as *moral*, and not classify some as *epistemic* (and hence directly supplementing existing epistemic work) and some as moral (opening a new avenue of critique)? It seemed to me that the harm to fairness (unfair of the community to tolerate some of the QRPs discussed because it puts those who do not engage in these QRPs at a disadvantage) is the only harm that seems to have no substantial epistemic component.

 Third, and perhaps obvious, it seems to me that some of these practices are only questionable if *not* disclosed, e.g., for Accountability, these detailed timelines are developed (I presume) to the best of the applicant’s ability, and if the applicant is successful, they need to be adjusted (perhaps in consultation with the funder) when unforeseen circumstances happen, or things otherwise turn out differently. Double-dipping is also a practice that need not be harmful, provided it is acknowledged to the funder.

**Smaller remarks: **

- “Since low reliability implies low validity,” (p. 3) is not correct, it *could* be the other way around, but I believe the rest of the sentences then don't hold anymore or need some revisions.
- “These epistemic problems would be acceptable if the costs… were relatively low.” seems odd to me, I think the scientific community and the public at large should demand a system that gets them the truest possible knowledge and that if you end up with peer review that gets you non-innovative work and happens by luck only, then the cost aren’t the greatest concern…

Is the topic of the opinion article discussed accurately in the context of the current literature?

Partly

Are arguments sufficiently supported by evidence from the published literature?

Partly

Are all factual statements correct and adequately supported by citations?

Yes

Are the conclusions drawn balanced and justified on the basis of the presented arguments?

Partly

Reviewer Expertise:

My area of interest is research integrity, in addition, I have a background in philosophy, psychology and epidemiology.

I confirm that I have read this submission and believe that I have an appropriate level of expertise to confirm that it is of an acceptable scientific standard, however I have significant reservations, as outlined above.

## References

[1] : Prevalence of Research Misconduct and Questionable Research Practices: A Systematic Review and Meta-Analysis. *Science and Engineering Ethics* .2021;27(4) : 10.1007/s11948-021-00314-9 10.1007/s11948-021-00314-9 34189653

# Response

**Published:** 2021-11-30

**Authors:** Stijn Conix

**Affiliations:** Catholic University of Leuven, Belgium

**Thank you for reviewing the paper, and for your very helpful comments -- they point out some of the issues we had been discussing at length before, and had difficulties resolving. We respond to your comments below.**

 REVIEWER:

 Is the topic of the opinion article discussed accurately in the context of the current literature?

 There is an increasing amount of research that investigates questionable research practices, a recent systematic review might provide some points the authors can connect to: Xie, Y., Wang, K., & Kong, Y. (2021). Prevalence of Research Misconduct and Questionable Research Practices: A Systematic Review and Meta-Analysis. Sci Eng Ethics, 27(4), 41.

**Thank you! As we think this might also be interesting for readers, we’ve added a line and this reference in the first paragraph of the paper.**

 REVIEWER: Are the conclusions drawn balanced and justified on the basis of the presented arguments?

 Most of the authors’ main conclusions are balanced and justified, but I find part of their concluding statements unjustified. In particular, “In fact, our assessment includes many of the ‘cardinal sins’ against research integrity: self-plagiarism (in the form of double-dipping), taking credit for someone else’s work (in cases where junior researchers write applications for their senior colleagues) and, potentially, falsification and fabrication (in cases where scientific results are adjusted to conform to promises made in the grant application).” p. 8 seems unjustified. The ‘cardinal sins’ indeed include plagiarism, but not *self*-plagiarism, which in various CoC is explicitly separated from plagiarising others, see e.g., Dutch CoC. Taking credit for someone else’s work is guest authorship, which is related to plagiarism but often not considered a cardinal sin. In the article, the authors talk about making claims in grant applications that the grant requesters may not be able to justify, but they don’t go in-depth into the pressure put on researchers *after* having received a grant, where these cases of potential falsification and fabrication should then supposedly happen. Whether the successful applicants do this seems an empirical claim to me, and one that seems rather unlikely.

**We agree that our statement about the cardinal sins was overstated and not fully justified, and have toned down this part of the paper. We now state (in that same paragraph, start of ‘discussion and conclusions’) that while many of the practices that we discuss may seem like rather minor offenses, some are unquestionably problematic and serious, such as ghost authorship (which we think is more pressing than guest authorship in the context of funding) and double dipping. This is sufficiently strong for the point we want to make.**

 REVIEWER: I have three suggestions. First, a substantial portion of the critique seems focused on the competition that is inherent in peer-review project funding, not so much the peer-review project funding system per se. The competition seems to do the heavy lifting in increasing the likelihood of researchers or reviewers engaging in questionable behaviour (at least for parts of Accountability, Honesty and Impartiality). I am aware that the authors acknowledge how PRPF is a case of organised competition, but it seems to me that PRPF in a situation of medium competition (instead of contemporary excessive competition) would be associated with less questionable behaviour or would at least be a much weaker incentive. It would be helpful to read how the authors see this, because the topic of competition has been discussed by various research integrity scholars (a great example is the paper by Anderson and colleagues from 2007).

**This is an interesting suggestion. We agree that strong competition is not inherent to PRPF, and that lowering competition (i.e. higher success rates) would lower the incentives to cut corners, and thus might lower some of the problems. However, as long as there is some competition -- which is inevitable in PRPF --  there will be an incentive to engage in some of the practices we discuss. How strong these are is unclear, as it may well be that those who don’t get funding are even worse off than before (for instance because their failing becomes more salient), and might be more likely to engage in questionable research practices.**

**Thus, the precise effect of increasing success rates is hard to predict, and it is far from clear that most of the problems that we discuss would disappear. We therefore decided to focus on the current situation and assess its problems.**

**We do think it is important to mention this point, and now do this in section 2 (second and third paragraphs), where we introduce PRPF. We now clearly state that currently PRPF is highly competitive, and have added an endnote saying that this high level of competition is not inherent to PRPF.**

 REVIEWER: My second suggestion is that although the authors acknowledge the inevitable overlap of epistemic and moral norms, the harm resulting from the questionable behaviours (QRPs) described seems primarily epistemic. I wonder why the authors hold on to framing their arguments as moral, and not classify some as epistemic (and hence directly supplementing existing epistemic work) and some as moral (opening a new avenue of critique)? It seemed to me that the harm to fairness (unfair of the community to tolerate some of the QRPs discussed because it puts those who do not engage in these QRPs at a disadvantage) is the only harm that seems to have no substantial epistemic component.

**We think that even fairness has a substantial epistemic component (see recent work on the epistemic importance of so-called non-epistemic values, e.g. this paper, and this book chapter), and agree that it is also the case for the other values we discuss. In science, it is difficult to disentangle these two dimensions, and perhaps not always helpful. What matters most is that we highlight issues that have not been recognised before, regardless of whether they are called epistemic or moral.**

**The reason why we explicitly frame them as moral is that, unlike other work on this topic that focuses solely on the epistemic dimension, we start from the fact that these behaviors are violations of codes of conduct. These codes explicitly state that they want to ‘guide researchers in their work as well as in their engagement with the practical, ethical and intellectual challenges inherent in research.’ (see European code of conduct for research integrity cited in the paper). Thus, while clearly also epistemic, these codes claim to have a moral dimension. In addition (but one doesn’t need to buy this to agree with what we state in the paper), we think that many of these behaviours (e.g. not being honest) would be wrong even if they wouldn’t do any epistemic harm.**

**We already had a paragraph on this issue in the paper, and have added a couple of sentences in that paragraph (end of section 3) to make it more explicit.**

 REVIEWER: Third, and perhaps obvious, it seems to me that some of these practices are only questionable if *not* disclosed, e.g., for Accountability, these detailed timelines are developed (I presume) to the best of the applicant’s ability, and if the applicant is successful, they need to be adjusted (perhaps in consultation with the funder) when unforeseen circumstances happen, or things otherwise turn out differently. Double-dipping is also a practice that need not be harmful, provided it is acknowledged to the funder.

**We agree that some of the problems can be diminished or removed by proper disclosure methods. However, even if it solves or alleviates some problems, it leaves many other problems unaddressed. In addition, we worry that for some problems proper disclosure methods are difficult to design. For example, uncertainty about evaluations of reviewers could be disclosed, but there are likely large differences between the ways different reviewers judge and represent their own uncertainty, and there is inevitably meta-uncertainty about their uncertainty. This would make the disclosed uncertainty very difficult to use, and, thus, part of the problem would remain.**

**We do think that this is a valuable suggestion, and have added it to the paragraph on ‘minor changes’ to PRPF (start of the discussion and conclusions section).**

 REVIEWER:  “Since low reliability implies low validity,” (p. 3) is not correct, it could be the other way around, but I believe the rest of the sentences then don't hold anymore or need some revisions.

**This now reads ‘Since low reliability is typically associated with low validity…’**

 REVIEWER: “These epistemic problems would be acceptable if the costs… were relatively low.” seems odd to me, I think the scientific community and the public at large should demand a system that gets them the truest possible knowledge and that if you end up with peer review that gets you non-innovative work and happens by luck only, then the cost aren’t the greatest concern…

**We agree that this statement makes a value-judgement that many readers might not share. We’ve toned down the sentence: ‘These epistemic problems might be justifiable if the costs…’**

# Peer review: Reviewer response for version 1

**DOI:** 10.5256/f1000research.77582.r99418

**Published:** 2021-11-12

**Authors:** Lambros Roumbanis

**Affiliations:** 1 Stockholm Centre for Organizational Research (SCORE), Stockholm University, Stockholm, Sweden

**Comments by Roumbanis to *Conix, De Block and Vaesen* (2021) “Grant writing and grant peer review as questionable research practices”**

 The critical approach that you have chosen for the present paper provides the reader with a good picture of what really is at stake in grant peer review. By demonstrating how the system of competitive funding prompts ethically questionable behaviors and opportunistic strategies, I believe you hit one of the two Achilles heels of the allocation system, the ethical (the other being the epistemological). If the distribution of research opportunities within the academic communities not only generates demoralized scholars, but also ethically questionable practices, then this seems to be a case of a “tragedy of the commons.” I truly sympathize with your effort to shed new light on how the incentives of peer-review project funding (PRPF) leads to undesirable social outcomes. Your paper is well-written and perfectly clear in its general line of argumentation. I have, however, four comments that I would like to share. Hopefully, you will find them relevant for your ongoing meta-scientific investigation.

        

 First, the Mertonian norms of science could be an important starting point for a general discussion about the ethical values that guide or at least ought to guide research. There have been a number of critical responses regarding the validity of Merton’s theoretical claims and some scholars have presented new alternative views. In any case, I interpreted your analysis of the codes of conduct (CoCs) as an implicit elaboration of Merton’s thesis about “the Matthew effects,” which you explicitly mention in the paper. In that sense, I believe your investigation makes a fine contribution to the ongoing dialogues within modern sociology of science. Yet my point is, that perhaps you should relate more explicitly to Merton for reasons of contextualization. Your study may also benefit theoretically if you dig deeper into these discussions (see e.g., Knuuttila 2012; Bielinski and Tomczynska 2019; Grundmann 2013; Stehr 2018). As I see it, when reading Merton’s own work, there certainly exists a tension between the “ethos of science” and the “organization of science” – a tension between the ideals and the realities of modern science. You show in your study how the ethos of science has been violated by the current competitive funding regimes in a way that adds a new problematic dimension to scientific progress.

 From a retrospective analysis, Serrano Velarde (2018) demonstrated how “the way we ask for money” have changed dramatically over the past hundred years in Germany. She illustrates this change in her study about the institutionalization of grant writing practices by showing a picture of Noble laureate in medicine Otto Warburg’s application from 1921, which contains only one short sentence saying “Ich benötige 10 000 (zehntausend) Mark” (in English: “I need 10.000 Mark”). This is a remarkable contrast with how a proposal must be composed today to have a chance of being funded.

              

 

    (Fig.1 from Serrano Velarde 2018, p.90)

 Second, when I read your analysis of the scientific codes of conducts (CoCs), I was a bit surprised by the absence of concepts like *solidarity* and *generosity*, especially regarding the issue of “greedy researchers” that apply for (and receives) more funding than they really need. Why do the rules of the funding system allow researchers to get their hands on several grants at the same time? As we know, researchers do not only need to have talent and the right kind of merits, they also need valuable connections and luck in order to succeed professionally. Here, one could possibly argue for the importance of *solidarity as fairness* from a modified Rawlsian framework, and thereby view generosity as a fundamental virtue for *a future ethics of research funding*. No matter who we are as researchers or what we have achieved at a certain point, solidarity and generosity always ought to rule. There will always be competition and disagreements between scholars, this is unavoidable; yet we should try to replace the disastrous competition for economic survival in academic science, “the war of all against all” ( *bellum omnium contra omnes*) with the traditional Greek ethical ideal of an honest “friendly rivalry” (ευγενής άμιλλα). Furthermore, if we seriously consider using another type of distribution mechanism than peer review (e.g., lottery or baseline funding), then our view of what we regard as just and fair in science, should perhaps also include the two aforementioned concepts. Now, I know that you acknowledged the possibility of alternative categorizations in your paper, and I agree that there must be overlaps between some of the values involved. For sure, the same thing goes for solidarity and generosity in relation to honesty, impartiality, responsibility and fairness.

 Third, relying on recommendations based on Artificial Intelligence (AI) could also be mentioned as a radical proposal for allocating resources. Highly sophisticated and well-designed AI system with the capacity to evaluate a large number of different academic merits and values, identify promising patterns and probabilities for scientific breakthroughs, without the influence of human biases, could perhaps be a more rational and fair method in the future. Obviously, this kind of proposal needs to be properly scrutinized from an ethics of AI perspective – the risks of instead introducing algorithmic biases and discriminatory treatment in academic science must be seriously considered and tested before AI can be implemented as a decision-making technology. Still, you might want to mention AI in your paper together with lotteries and baseline funding as new possible alternatives to traditional peer review.

 Finally, you analyze both the virtues and vices of the current funding regime. However, other important socio-ethical dilemmas that I myself have been thinking about are, for example, organizational side-effect like *symbolic violence*. In an observational study that I conducted some years ago (Roumbanis 2019) I analyzed the subtle form of power that senior faculty members sometimes tend to exercise on junior scholars when they hold lectures on grant writing and “the art of getting funding”. The issue of researchers having to navigate in relation to different “academic value spheres” (Ekman 2017) also demonstrates how difficult it can be for researchers to embody the codes of conduct, especially for junior scholars to avoid opportunistic actions in precarious situations. These studies, I believe, fits rather well with your own main narrative in the present paper. In addition, the issue of *hypocrisy*, generated in contemporary organizations partly because of the increasing number of conflicting demands and expectations, could also be taken into account here (Brunsson 2019). Both symbolic violence and hypocrisy could in fact be part of a general description of the problematic side-effects of the organization and culture of the current system of peer-review project funding.

 To conclude: this is an inspiring and interesting paper about the ethical dilemmas in the funding system that currently dominates in most OECD countries. You have indeed highlighted some of the most notorious problems that many of us are witnessing today in academic science. In my view, this is a subject that deserves and is in great need, of further investigation in the increasingly complex research landscape. A small suggestion would be to put the word “ethically” in the title, that is, “Grant writing and grant peer review as ethically questionable research practices.”

 Sincerely,

 Lambros

Is the topic of the opinion article discussed accurately in the context of the current literature?

Yes

Are arguments sufficiently supported by evidence from the published literature?

Yes

Are all factual statements correct and adequately supported by citations?

Yes

Are the conclusions drawn balanced and justified on the basis of the presented arguments?

Yes

Reviewer Expertise:

Sociology of Science; Science and Technology studies (STS); Evaluation studies; Theory of Science; Sociology of Judgment and Decision-making.

I confirm that I have read this submission and believe that I have an appropriate level of expertise to confirm that it is of an acceptable scientific standard.

## References

[1] : The Ethos of Science in Contemporary Poland. *Minerva* .2019;57(2) : 10.1007/s11024-018-9365-1 151-173 10.1007/s11024-018-9365-1

[2] : The Organization of Hypocrisy: Talk, Decisions and Actions in Organizations (3rd edition). *Business School Press, Copenhagen* .2019;

[3] : A history of vocational ethics and professional identity: How organization scholars navigate academic value spheres. *Human Relations* .2017;70(4) : 10.1177/0018726716660370 461-487 10.1177/0018726716660370

[4] : “Climategate” and The Scientific Ethos. *Science, Technology, & Human Values* .2013;38(1) : 10.1177/0162243911432318 67-93 10.1177/0162243911432318

[5] : Contradictions of Commercialization: Revealing the Norms of Science?. *Philosophy of Science* .2012;79(5) : 10.1086/667844 833-844 10.1086/667844

[6] : Symbolic Violence in Academic Life: A Study on How Junior Scholars are Educated in the Art of Getting Funded. *Minerva* .2019;57(2) : 10.1007/s11024-018-9364-2 197-218 10.1007/s11024-018-9364-2

[7] : The Way We Ask for Money… The Emergence and Institutionalization of Grant Writing Practices in Academia. *Minerva* .2018;56(1) : 10.1007/s11024-018-9346-4 85-107 10.1007/s11024-018-9346-4

# Response

**Published:** 2021-11-30

**Authors:** Stijn Conix

**Affiliations:** Catholic University of Leuven, Belgium

**Thanks for reading our paper, and for your insightful and charitable comments! They illustrate that there is far more to be said about this topic than we managed to cover in this paper. We’ve made various changes on the basis of your comments, and think they have improved the paper. We respond to your comments point by point below.**

 REVIEWER: First, the Mertonian norms of science could be an important starting point for a general discussion about the ethical values that guide or at least ought to guide research. There have been a number of critical responses regarding the validity of Merton’s theoretical claims and some scholars have presented new alternative views. In any case, I interpreted your analysis of the codes of conduct (CoCs) as an implicit elaboration of Merton’s thesis about “the Matthew effects,” which you explicitly mention in the paper. In that sense, I believe your investigation makes a fine contribution to the ongoing dialogues within modern sociology of science. Yet my point is, that perhaps you should relate more explicitly to Merton for reasons of contextualization. Your study may also benefit theoretically if you dig deeper into these discussions (see e.g., Knuuttila 2012; Bielinski and Tomczynska 2019; Grundmann 2013; Stehr 2018). As I see it, when reading Merton’s own work, there certainly exists a tension between the “ethos of science” and the “organization of science” – a tension between the ideals and the realities of modern science. You show in your study how the ethos of science has been violated by the current competitive funding regimes in a way that adds a new problematic dimension to scientific progress.

 From a retrospective analysis, Serrano Velarde (2018) demonstrated how “the way we ask for money” have changed dramatically over the past hundred years in Germany. She illustrates this change in her study about the institutionalization of grant writing practices by showing a picture of Noble laureate in medicine Otto Warburg’s application from 1921, which contains only one short sentence saying “Ich benötige 10 000 (zehntausend) Mark” (in English: “I need 10.000 Mark”). This is a remarkable contrast with how a proposal must be composed today to have a chance of being funded.

**We now acknowledge in the paper that CoCs are typically based on Merton’s regulative ideals. And we have added that: “This is not to say that the Mertonian norms haven’t been criticized (See Knuuttila 2012 for an overview). For one, there seems to be a disconnect between the norms and actual scientific practice, especially in the context of commodified science. In this sense, the ethos of science conflicts with the organization of science. In fact, our arguments about PRPF illustrate that the organization of science indeed is difficult to bring into accord with the regulative ideals formulated by Merton.”**

 REVIEWER: Second, when I read your analysis of the scientific codes of conducts (CoCs), I was a bit surprised by the absence of concepts like solidarity and generosity, especially regarding the issue of “greedy researchers” that apply for (and receives) more funding than they really need. Why do the rules of the funding system allow researchers to get their hands on several grants at the same time? As we know, researchers do not only need to have talent and the right kind of merits, they also need valuable connections and luck in order to succeed professionally. Here, one could possibly argue for the importance of solidarity as fairness from a modified Rawlsian framework, and thereby view generosity as a fundamental virtue for a future ethics of research funding. No matter who we are as researchers or what we have achieved at a certain point, solidarity and generosity always ought to rule. There will always be competition and disagreements between scholars, this is unavoidable; yet we should try to replace the disastrous competition for economic survival in academic science, “the war of all against all” (bellum omnium contra omnes) with the traditional Greek ethical ideal of an honest “friendly rivalry” (ευγενής άμιλλα). Furthermore, if we seriously consider using another type of distribution mechanism than peer review (e.g., lottery or baseline funding), then our view of what we regard as just and fair in science, should perhaps also include the two aforementioned concepts. Now, I know that you acknowledged the possibility of alternative categorizations in your paper, and I agree that there must be overlaps between some of the values involved. For sure, the same thing goes for solidarity and generosity in relation to honesty, impartiality, responsibility and fairness.

**We agree that a classification of values/norms that includes generosity and/or solidarity would have made a lot of sense, and now mention these two norms as good alternatives to our classification where we motivate our choice of categories (at the end of section 3). By explicitly mentioning them, we hope to increase the chance that any future work on the ethics of research funding considers them explicitly.**

**As you also mention, the choice of categories is a bit arbitrary, and we think that what matters most in our paper is that we find a sensible home for all the morally problematic behaviors relating to research funding. To avoid having to drastically change the paper structure, we have therefore kept the classification of norms the same as it was before. Overfunding and missing the funding sweet spot are clearly transgressions of norms like generosity and solidarity, but we think that these behaviours can also be framed as violations of responsibility, fairness and honesty.**

 REVIEWER: Third, relying on recommendations based on Artificial Intelligence (AI) could also be mentioned as a radical proposal for allocating resources. Highly sophisticated and well-designed AI system with the capacity to evaluate a large number of different academic merits and values, identify promising patterns and probabilities for scientific breakthroughs, without the influence of human biases, could perhaps be a more rational and fair method in the future. Obviously, this kind of proposal needs to be properly scrutinized from an ethics of AI perspective – the risks of instead introducing algorithmic biases and discriminatory treatment in academic science must be seriously considered and tested before AI can be implemented as a decision-making technology. Still, you might want to mention AI in your paper together with lotteries and baseline funding as new possible alternatives to traditional peer review.

**This is a good suggestion, thanks! We now briefly mention (in the ‘discussion and conclusions’ section, paragraph starting with ‘A final, more radical option…’) that while at the moment there are no AI-systems that work well enough (and work on this topic is still very scarce, see reference in the paper), this might well be a viable alternative in the future as long as we remain on our guard  for possible algorithmic biases.**

 REVIEWER: Finally, you analyze both the virtues and vices of the current funding regime. However, other important socio-ethical dilemmas that I myself have been thinking about are, for example, organizational side-effect like symbolic violence. In an observational study that I conducted some years ago (Roumbanis 2019) I analyzed the subtle form of power that senior faculty members sometimes tend to exercise on junior scholars when they hold lectures on grant writing and “the art of getting funding”. The issue of researchers having to navigate in relation to different “academic value spheres” (Ekman 2017) also demonstrates how difficult it can be for researchers to embody the codes of conduct, especially for junior scholars to avoid opportunistic actions in precarious situations. These studies, I believe, fits rather well with your own main narrative in the present paper. In addition, the issue of hypocrisy, generated in contemporary organizations partly because of the increasing number of conflicting demands and expectations, could also be taken into account here (Brunsson 2019). Both symbolic violence and hypocrisy could in fact be part of a general description of the problematic side-effects of the organization and culture of the current system of peer-review project funding.

**It is true that both hypocrisy and the kind of symbolic violence that you describe are part of many of the problems that we discuss: many of the practices are common and generally tolerated, and this creates a culture where adhering to codes of conduct is sometimes difficult for particular groups of researchers. Because we think that this problem is particularly pressing for junior scholars, we now mention this in the section on fairness, in the context of tolerating misconduct.**

 REVIEWER: To conclude: this is an inspiring and interesting paper about the ethical dilemmas in the funding system that currently dominates in most OECD countries. You have indeed highlighted some of the most notorious problems that many of us are witnessing today in academic science. In my view, this is a subject that deserves and is in great need, of further investigation in the increasingly complex research landscape. A small suggestion would be to put the word “ethically” in the title, that is, “Grant writing and grant peer review as ethically questionable research practices.”

**As the other reviewer highlighted that our points are epistemic as well as ethical, we think it better to keep the title as it is, and focus on the point that these practices are problematic.**
